# 우울한 임신 여성의 화상 인지행동치료 프로그램 참여 경험: 탐색적 질적연구

**DOI:** 10.4069/kjwhn.2022.11.28

**Published:** 2022-12-29

**Authors:** Eunjoo Lee, Mijung Kim, Youngsuk Park

**Affiliations:** 1 Department of Nursing, Kyungnam University, Changwon, Korea; 1 경남대학교 간호학과; 2 Department of Nursing, Changshin University, Changwon, Korea; 2 창신대학교 간호학과; 3 College of Nursing, Taegu Science University, Daegu, Korea; 3 대구과학대학교 간호대학

**Keywords:** Cognitive behavioral therapy, Depression, Pregnancy, Qualitative research, 인지행동치료, 우울, 임신, 질적연구

## Abstract

**Purpose:**

This study explored the experiences of pregnant women with depressed mood participating in a group cognitive behavioral therapy (CBT) program using video communication, based on Beck’s cognitive theory.

**Methods:**

The participants were six pregnant women out of 13 women who had participated in an 8-session group CBT program using video communication for women with depressed mood (Edinburgh Postnatal Depression score of ≥9). Data were collected from February 20 through March 25, 2021. In-depth individual interviews were conducted through a video conferencing platform at 1 month post-baseline. Thematic analysis was done.

**Results:**

Three themes, 10 subthemes, and 38 concepts were derived from experiences of participating in the 4-week group CBT program (twice a week). The first theme, entitled “continuing realization” had subthemes of “a negative and instable self,” “a selfish judgment that excludes others,” and “a strong belief in self-control.” The second theme, entitled “attempt to change for restoration” had subthemes of “shift to rational thinking,” “freedom from suppressed beliefs,” “tolerance of other people,” and “courage for self-expression.” The third theme, entitled “departure for a positive life,” had subthemes of “emotional healing,” “faith in oneself,” and “reestablishing the criteria for happiness.”

**Conclusion:**

Pregnant women with depressed mood expressed that continuing realizations and attempts to change supported their transition toward a positive direction of healing. Thus, they were able to change their distorted thinking into rational thinking through CBT using video communication. These findings support the use of group CBT using video communication with pregnant women who have depressed mood.

## Introduction

임신 중 우울증은 임신 여성의 약 7%가 경험하는 빈번한 건강문제로[1], 절망감, 불안, 슬픔, 죄책감, 불면 혹은 수면 과다, 과식 혹은 식욕 상실, 집중력 및 기억력 저하, 과민 또는 안절부절 등의 증상을 동반한다[2]. 이 가운데 수면 및 식욕 문제, 에너지 수준 저하와 같은 일부 증상들은 임신 증상과 유사하기 때문에 임부가 우울 증상임을 자각하지 못할 수도 있다[1].

우울증은 임신 여성뿐만 아니라 자녀와 가족에게까지 치명적인 결과를 초래할 수 있으므로, 조기에 관리할 필요가 있다[3]. 치료하지 않을 경우 태아를 위한 영양 섭취가 어렵고 활동 에너지가 없으며[1], 자살충동이 높아지고 산후정신병으로도 발전할 수 있다[4]. 우울증 선별검사는 우울증 진단을 대신할 수는 없지만, 추가 평가에 대한 필요성을 확인할 수 있어 유용한 검사방법이다[5]. 그러나 선별검사에서 우울증 위험이 발견되더라도 대부분의 임산부는 재평가를 하거나 치료를 받지 않는 것으로 보고된다[4].

우울증은 그 정도의 심각성에 따라 정신요법이나 항우울제와 같은 전문치료 이외에도 심리요법이 고려된다[1]. 인지행동치료(cognitive behavioral therapy, CBT)는 우울증 치료에 효과적인 방안으로 널리 알려져 있으며, 임신과 출산을 포함한 주산기 우울증 치료에도 널리 사용되고 있다[3]. CBT는 개인의 사고가 자신의 감정과 행동에 영향을 미치고, 행동은 감정과 사고에 영향을 미친다는 데에 기초한다[6]. 우울증 치료에 있어서 인지이론 적용의 목적은 부적응적 사고의 패턴을 다루고 인지를 재구조화함으로써 부정적 정서반응과 행동을 줄이는 데에 있다[7].

한편, 우울한 임신 여성의 경우 대인관계에서 고립되거나 외부활동을 기피할 수 있어 대면치료에 어려움이 있다[7]. 게다가 최근 코로나바이러스감염증-19 (coronavirus disease 2019, COVID-19) 확산으로 인하여 외부활동이 제한되면서 우울 수준이 높은 임신 여성들을 위한 최적의 치료방안이 필요해진 상황이다. 웹 기반 CBT는 온라인 플랫폼으로 전달되며, 텍스트를 기반으로 정보나 자료를 활용하여 운영된다[8]. 우울증을 포함한 정신건강 문제뿐만 아니라 신체질환을 가진 대상자들에게도 대면 CBT만큼 효과적이지만[9], 치료자와 내담자 간의 상호작용 및 지원이 부족하다는 단점이 있다[10]. 그러나 화상회의의 경우 치료를 위한 자료 공유가 쉽고 접근성이 좋으며[11] 서로 소통이 가능하기 때문에 언어적 의사소통을 통한 긍정적인 치료관계가 가능하다[12]. 따라서 화상 CBT 프로그램이 우울한 임신 여성의 사고와 감정, 그리고 행동에 어떠한 변화를 일으키는지 확인한다면 임신 중 우울증 관리를 위한 새로운 전략을 제공할 수 있을 것이다.

웹 기반 CBT 참여 경험에 관한 국외 질적연구는 몇 편이 있었다[13,14]. Berg 등[13]의 연구는 주요 우울증 진단을 받은 청소년을 대상으로 인터넷 CBT에서 배운 지식의 습득과 활용에 초점을 맞추었고, Chan 등[14]의 연구는 우울증 대상자의 불면증 치료를 위한 임상 치료와 인터넷 CBT 병행의 효과에 중점을 두었다. 국내의 경우 공황장애[15], 강박장애[16], 도박중독[17] 등에 적용한 대면 CBT 참여 경험에 관한 질적연구가 있었으나 화상 CBT 참여 경험과 임신 여성을 대상으로 한 질적연구는 없었다.

우울 수준이 높은 임신 여성에게 적용한 화상 CBT 선행연구[18]에서 참여자의 부정적 감정과 사고가 유의한 감소를 보였으나, 이는 양적연구의 계량화된 값이므로 총체적인 상황에서의 변화를 이해하기가 어려웠다. 이에 본 연구는 우울한 임신 여성들이 화상 CBT 프로그램에 참여한 경험을 인지 변화의 측면에서 심층적으로 탐색함으로써, 화상 CBT의 효과를 이해하고 우울한 임신 여성을 관리하기 위한 중재방안을 마련하는 기초자료를 제공하고자 한다.

본 연구의 목적은 Beck의 인지이론[3]에 근거하여 화상 CBT 프로그램에 참여한 우울한 임신 여성의 경험을 탐구하는 데에 있다.

## Methods

Ethics statement: This study was approved by the Institutional Review Board of Kyungnam University (No. IRB-1040460-A-2020-039). Informed consent was obtained from the participants.

### 연구 설계

본 연구는 우울한 임신 여성의 화상 CBT 프로그램 참여 경험을 이해하고 탐색하기 위한 질적연구이며, COREQ (Consolidated Criteria for Reporting Qualitative Research) 가이드라인에 따라 기술하였다[19].

### 연구 대상

본 연구에서는 연구 참여자 선정을 위해 목적적 표집을 실시하였다. 8회기의 화상 CBT 프로그램[19]에 참여했던 임신 18주–28주 사이의 임부 중, 본 질적연구의 목적을 이해하고 연구 참여에 자발적으로 동의한 자를 대상자로 선정하였다. 화상 CBT 프로그램의 참여자는 창원시 온라인 임신‧육아 커뮤니티인 ‘맘카페’ 회원으로, 에딘버러 산후우울 척도 (Edinburgh Postnatal Depression Scale) 점수가 9점 이상이었다. 에딘버러 산후우울 척도의 기준을 9점으로 선정한 이유는 보건복지부에서 이를 우울상담이 필요한 수준이라고 제시하고 있기 때문이다[20].

### 자료 수집 방법

본 연구에서 화상 CBT 프로그램은 창원시 온라인 임신‧육아 커뮤니티인 맘카페에 안내하였고, 프로그램 진행 기간은 2020년 10월부터 2021년 2월까지였다[19]. 총 3, 4인이 한 팀을 이루어 1회 당 80분(CBT 그룹 활동 50분, 과제 나눔 30분), 주 2회, 총 8회기를 진행하였다. 회차별 순서는 지난 회기 이후의 생활에 대해 참여자들과 공유하고, 매 회기의 주된 내용 학습, 활동 후 느낌 나누기, 과제 안내 순서로 진행하였다. 1회기는 ‘인지행동 산후우울 예방 프로그램 소개’를 통해 인지행동의 기본 개념과 참여자들을 소개하고, 2회기인 ‘긍정의 나 부정의 나’는 일상생활에서 일어나는 자신의 생각과 감정을 확인한다. 3회기 ‘스트레스 확인하기 1’은 자신의 평소 스트레스 정도를 확인하고, 4회기 ‘스트레스 확인하기 2’는 임신과 관련한 자신의 스트레스 내용을 확인한다. 5회기 ‘스트레스 대처하기’는 자신의 핵심 신념과 평소 자신의 스트레스 조절 효과를 확인하고, 6회기 ‘내 삶의 이야기’는 자신의 일상에서 나타나는 자동적 사고, 핵심 신념, 인지적 오류, 감정 확인을 통해 자신의 삶을 객관적으로 바라본다. 7회기 ‘갈등에 대한 변화’는 갈등 상황에서 자신이 사용하는 문제 해결 방식의 변화를 통한 효과성을 확인하고, 8회기 ‘변화하는 나의 삶 이야기’는 갈등 사건에 대한 가치를 재정의하는 과정을 통해 자신의 새로운 미래를 계획해 보도록 하였다.

총 8회의 화상 CBT 프로그램 참여를 통해 우울, 자동적 사고 및 역기능적 태도의 점수가 유의하게 감소한 것을 확인할 수 있었다[18]. 본 연구는 화상 CBT 프로그램 종료 1개월 시점에서 프로그램에 참여했던 13명에게 유선으로 연구의 목적, 방법 등을 설명하고, 참여 의사를 밝힌 6명에게 동의서를 받은 뒤 자료 수집을 위한 심층면담을 실시하였다. 선행 연구들[12,16]에서는 자료 수집 기간이 프로그램 진행에서부터 종료 후 12개월 이내였으며, 본 연구에서는 출산 이후 심층면담에 집중하기 어려울 것을 감안하여 프로그램 종료 후 1개월에 면담을 실시하였다.

본 질적연구의 자료 수집은 2021년 2월 20일에서 3월 25일까지 진행되었다. 면담을 시작하기 전 면담 내용의 녹음에 대해 허락을 받았으며, COVID-19 위기 상황에서 대면이 어려워져 참여자가 원하는 시간에 맞추어 화상으로 심층면담을 진행하였다. 면담 횟수는 1인당 1–2회였고, 면담 시간은 1회당 약 50분에서 1시간 30분이 소요되었다. 면담 후에는 소정의 사례비(25,000원)를 지급하였다.

면담은 반구조화된 질문 형식으로 진행하였다. 주요 면담 질문으로는 “화상회의 CBT 프로그램 참여 동안 어떤 경험을 하였습니까?”, “프로그램 참여 전 생각과 행동은 어떠했습니까?”, “프로그램 참여 후 무엇이 달라진 것 같습니까?”, “프로그램 참여 후 생활 속에서 어떤 변화를 경험했습니까?” 등이었다. 면담 시 참여자의 언어적, 비언어적 표현 등을 주의 깊게 관찰하고 메모하였다. 각 면담이 끝난 후에는 연구자와 연구보조원이 녹음 파일을 들으면서 참여자의 표현 그대로 필사를 하였으며, 추가 면담이 필요했던 3명의 경우 1회, 10–20분 정도 유선으로 자료를 수집하였다. 연구 참여자의 민감 정보인 녹음 파일과 필사본은 참여자의 정보를 코드화하여 저장하여 익명성이 보장된다는 것과 모든 자료는 연구 종료 후 폐기될 것임을 설명하였다.

### 자료 분석

자료 분석은 질적분석을 위한 기본방법으로 정성적 자료 분석에 유용한 주제분석(thematic analysis) [21] 6단계를 사용하였다. 1단계는 자료에 친숙해지는 단계로 필사된 면담 내용을 전체적으로 반복해서 읽으면서 의미 있는 부분에 밑줄을 그었다. 2단계는 초기 코드를 생성하는 단계로 친숙화 작업 후 자료에서 의미가 있는 공통된 특성을 코드화하였고, 각 코드와 관련된 자료를 모았다. 3단계는 주제 찾기 단계로 코드를 개념과 대조하고, 개념과 관련된 모든 자료를 수집하였다. 4단계는 주제 확인 단계로 도출된 개념이 전체 자료와 부합되는지 확인하고 주제도를 통해 각 주제가 어떻게 개념화되어 서로 관련되는지 검토하였다. 5단계는 주제를 정의하고 명명하는 단계로 전반적인 내용과 연관성을 두고 주제의 본질을 확인한 후 지속적 분석을 통해 주제를 명명하였다. 6단계는 보고서를 작성하는 단계로 연구질문이나 문헌과의 관련성을 검토하고 각 주제들이 내포하는 의미를 기술하였다.

### 연구의 엄격성 확보 및 연구자 준비

#### 연구의 엄격성 확보

본 연구에서 연구의 엄격성을 확보하기 위해 Lincoln과 Guba [22]가 제시한 평가기준인 신빙성, 전이성, 의존가능성, 확증가능성의 측면에서 연구의 신뢰도와 타당성을 높이고자 하였다. 연구자들은 신빙성을 확보하기 위해 연구 참여자에게 연구의 분석과 결과물에 대해 정확성을 확인할 것을 요청하여 왜곡된 내용이 없는지, 표현과 기술이 정확한지 등을 검토하였다. 전이성은 독자들에게 참여자의 면담 내용을 가능한 풍부하고 생생하게 기술하고자 하였고 프로그램, 전형적인 사례, 연구 대상자에 대해 묘사하였다. 의존가능성은 연구자 간에 일관된 결과가 도출되도록 수차례의 검토와 논의 과정을 거쳤으며, 확증가능성은 질적연구의 경험이 풍부한 교수 2인에게 분석자료와 연구결과에 대해 검토 받았다.

#### 연구자 준비

본 연구에서 제 1저자는 질적연구 특강이나 워크숍 등에 참여하여 질적연구 설계, 질적내용 분석, 현상학, 근거이론 등 질적연구방법론을 꾸준히 익혀왔다. 현상학적 연구 방법론을 이용한 질적연구를 학회지에 여러 편 게재한 경험이 있으며, 주산기 여성의 우울에 관한 연구를 10년간 수행해 오고 있다. 교신저자는 정신간호학 교수이며, 정신건강복지센터에서 조현병, 조기정신증, 자살 고위험군 아동청소년 등을 대상으로 인지행동치료를 실시한 경험이 있다. 또한 대학원 과정에서 질적연구방법론을 수강하였고, 질적연구방법론 워크숍에 참여하여 질적연구에 대한 기초를 다졌다. 공동 연구자 1인은 근거이론 연구로 박사 학위를 받고 질적연구방법론 워크숍, 학회 등에 꾸준히 참여하고 있으며, 근거이론방법을 이용한 질적연구를 학회지에 게재하였다.

## Results

본 연구의 참여자는 총 6명이었으며, 평균 연령은 34.3세(범위, 30–37세)였다. 현재 근무 중인 경우가 2명, 직업이 없는 경우가 3명, 휴직 상태인 경우가 1명이었다. 임신의 횟수는 평균 1.67회였으며, 제태연령은 평균 22주였고, 최소 18주에서 최대 28주였다. 임신 합병증이 있는 경우가 1명이었고, 가족 중 우울 진단을 받은 경우가 1명이었다. 우울 점수는 CBT 참여 이전의 경우 30점 만점에 평균 14.33점이었으며, CBT 종료 후에는 평균 5.5점이었다(Table 1).

본 연구에서 화상 CBT 프로그램[19]에 참여했던 우울한 임신 여성들의 경험을 탐색적으로 분석한 결과, 38개의 개념과 10개의 하위 주제, 3개의 주제가 도출되었다(Table 2).

### 화상 CBT 프로그램 참여 경험

#### 주제1. 알아차림의 연속

참여자들은 자기성찰의 사간을 통해 자신의 부정적 감정과 사고를 똑바로 알게 되었고, 이러한 것들로 인해 일상생활이 무기력하고 우울했음을 깨닫게 되었다. 지나친 열등감과 자신만 임신의 희생양이 된 것 같은 억울함에 휩싸여 스스로 외롭고 비참하게 만들었던 순간들을 반성하게 되었다. 이러한 감정과 사고는 자신의 삶에 부정적 영향을 미치기 때문에, 벗어나고 맞서야 함을 인식하게 되었다.

##### • 부정적이고 불안정한 자아

참여자들은 과거에도 부정적인 감정과 생각을 인식하곤 했지만 임신과 함께 자신이 더 부정적으로 변화되어가고 있음을 인식하게 되었다. 임신, 출산, 양육에 대한 걱정은 불안, 우울, 분노 등의 위태로운 감정으로 커져갔고 일상에서 빈번해졌다. 누구도 대신해 줄 수 없는 임신의 신체적 고통과 자녀 양육에 대한 막중한 부담감에 압도되어 감정의 불안정이 시작되었다고 회상했다. 타인과 비교하여 자신을 비하하고, 예상치 못한 부정적인 사건이 발생하면 일방적으로 자신에게 책임을 전가하는 습관이 있음을 알게 되었다.

입덧 때문에 아무것도 못하고 먹는 것도 힘들었어요. 내 몸은 이런데 아기를 케어하기 힘들고 남편하고 다투게 되면 감정적으로 휘둘리게 되었어요. 나만 왜 이렇게 고통스러울까? 우울한 생각이 들었어요. (참여자 1)

임신 후 몸이 너무 힘드니까 단단하지 못한 엄마라는 생각을 했어요. 엄마가 되어가는 내 모습이 약하고, 엄마로써 자격이 없다고 생각했어요. (참여자 3)

자궁에 피고임이 심해 누워서 생활을 했어요. 애기 상태가 나빠질 수 있다고 하니까 제가 몸 관리를 잘못해서 일이 발생하고, 다 내 잘못 같았어요. 그 때 겨울이었는데 세탁기가 얼어도 제 잘못이라고. 결빙 방지를 안 돌려 세탁기가 얼었구나 생각했어요. (참여자 6)

##### • 상대방을 배제한 이기적인 판단

참여자들은 상대방과의 관계에서 자신의 입장에서만 고집하여 올바른 판단을 하지 못하는 자신을 발견하게 되었다. 자신의 입장만 완고히 고수하여 상대방의 입장과는 다르게 자신을 희생하거나 상대방을 오해하게 만들었다. 자신이 늘 피해자이며, 상대방은 이기적이라는 편협한 사고방식에 문제가 있음을 반성하고, 상대방의 입장에서 생각하고 배려하는 것이 필요하다고 인식하게 되었다.

엄마 집까지 자가용으로 20분 정도 걸리는데, 엄마가 오라고 하면 내가 힘들지만 가지 않게 되면 좋은 딸과 멀어진다고 생각을 했어요. (참여자 1).

내 이야기에 토 다는 친구들이 있어요. 내 말을 왜 인정 안 해주고 부정을 하나, 나를 인정하지 않는구나 생각했어요. (참여자 3)

복잡한 업무인데, 일을 시키신 언니가 당연하게 시키고, 일이 반복되다 보니 기분이 나빠졌어요. 상황이 힘든데, 이런 나를 생각해주지 않는다고 생각이 들었어요. (참여자 6)

##### • 자신을 제어하는 강한 신념

참여자들은 자신에게 세상을 바라보는 견고하고 지배적인 관점이 있음을 깨닫게 되었다. 의식적이든 무의식적이든 자신을 억누르는 생각이 자신의 감정과 행동을 강하게 다스리며 삶의 전반을 조종하고 있었다. 이 생각을 고수하는 것이 자신을 지키는 것이라 믿었으며, 생각의 틀을 벗어나지 않으려고 전전긍긍하는 자신을 발견하게 되었다. 이러한 생각에 갇히게 되면 자신뿐 아니라 상대방도 불행하게 만들 수 있다고 생각하게 되었다.

남편도 가족이라고 생각 안하고, 피해 주면 안 된다. 내가 해결을 해야 하고, 스스로 해야 하고, 남편에게도 의논을 안 했던 것 같아요. (참여자 2)

경쟁사회에서 늘 잘해야 한다, 잘 하지 못하면 인정받지 못한다는 마음이 컸어요. (참여자 3)

사람에게 상처를 주면 안 된다는 가치관을 두고 사는 사람인데 그것을 무시하면서 계속 언니에게 상처만 주었어요. (참여자 6)

#### 주제2. 회복을 위한 변화 시도

자신의 사고에 집착하여 상황을 바라보는 것은 현실을 잘못 해석하게 만들어 판단능력을 떨어뜨린다는 사실을 깨닫고, 사고의 틀을 깨기 위한 연습을 일상생활에서 적용하려 하였다. 갈등 사건이 발생하거나 불편한 순간이 오면 CBT에서 배운 내용을 떠올리며 올바르게 생각하고 실천하려고 의식적으로 노력하였다. 과거와 달리 바람직한 결과를 향해 자신의 감정과 사고를 조절할 수 있게 되고, 상대방의 입장도 헤아려보려는 의지를 가지게 되었다.

##### • 합리적 사고로의 전향

참여자들은 과거 갈등 상황에서 감정에 휘둘려 행동했지만, 이제는 감정을 멈추고 사고하는 시간을 가지게 되었다. 타인과 적당한 거리를 유지하여 제 3자의 관점에서 상황을 인식하는 시각을 늘리게 되면서 마음의 여유가 생겨나고 유연하게 대처할 수 있게 되었다. 나쁜 감정이 갈등으로 연결되지 않도록 행동하기 전 합리적이고 긍정적인 이유를 찾게 되었고, 감정을 가라앉힐 수 있게 되었다. 부정적인 감정과 생각이 들 때마다 희망적이고 긍정적인 생각으로 자신의 선택을 바꾸어나가기 위해 애를 썼다.

입덧이 심한데 아는 동생은 입덧을 하지 않았다고 해서 약이 올랐어요. 나는 임신 체질이 아닌 것 같다고 생각이 들었어요. 그때 입덧은 호르몬 변화 때문에 일어나는 현상이라는 생각을 떠올리니, 마음이 편했어요. (참여자 1)

초음파를 보러 갔는데 아기 얼굴이 안 보였어요. 의사가 다시 보자고 할 줄 알았는데 다음에 보자고 하는 거예요. 화가 났는데, 애기가 안 보여주는 거니까 어쩔 수 없다고 생각하니까 마음이 편해졌어요. (참여자 3)

불편한 상황이 되면 그 길 위에 서 있게 되더라고요. 감정이 올라오는 순간 생각을 해야 된다고 생각하게 되었어요. (참여자 5)

##### • 억눌린 신념에서 벗어나기

참여자들은 ‘항상’, ‘반드시’, ‘꼭’ 등과 같이 나만의 규칙이라고 믿어왔던 사고의 테두리에서 빠져나오기 위해 노력하였다. 불편한 상황 속에서 자신을 지배했던 생각을 멈추고, 자신만의 논리를 내세워 그 생각에서 빠져나오려고 하였다. 문제나 갈등상황을 꼭 해결해야 된다는 강박적인 생각보다 있는 그대로를 받아들이려 했으며, 모든 것을 혼자 해결해야만 하고, 자신은 항상 잘 해야 한다는 생각에서 벗어나야 함을 알아가게 되었다.

힘들어하는 것을 인정하고 스트레스 받지 않으려고 하니까, 상황이 어쩔 수 없다고 인정하고 깨닫게 되니까 편안해졌어요. (참여자 1)

남편에게 도움을 청할 생각도 못하고 혼자서 해결하려고 했을 텐데, 수업 후 많이 바뀌어서 많이 편해진 것 같아요. (참여자 2)

잘 해야 한다는 강박이 있었는데 누구나 실수를 하는 것은 괜찮다고 생각해요. (참여자 4)

##### • 상대방에 대한 포용

참여자들은 과거나 현재의 갈등 상황에서 상대방의 입장이 되어 상대방의 마음을 공감하고 그때의 행동을 너그럽게 받아들이려고 하였다. 행동의 잘잘못을 가리기보다는 그럴만한 이유에서의 정당성을 부여하며 열린 마음으로 상대방과 그 상황을 이해해보려 하였다.

남편이 일을 그만두고 싶다고 했어요. 일을 해야 생활을 할 수 있는데 신랑의 입장에서 보면 출퇴근 시간이 길고, 잔업이 많아서 힘들 것 같아요. (참여자 1)

처음 아빠가 되다 보니 제 몸 상태나 뭘 해주어야 하는지 모르고, 애기가 생긴 것에 대한 기쁨은 있지만 평소대로 대해준 거 같아요. (참여자 3)

임용 준비를 그만 하라는 부모님을 만나고 싶었는데 끝까지 밀어붙이셨어요. 엄마가 나를 얼마나 안타까워하는지 생각을 안 해봤어요. 나는 사랑을 많이 받고 있는데... (참여자 4)

##### • 자기 표현의 용기

참여자들은 상대방의 입장을 들어보고, 서로의 요구를 확인하면서 합일점이 찾아지는 경험을 하였다. 과거에는 상황을 회피하면서 상대방을 원망하기 일쑤였지만 이제는 먼저 다가가 대화를 시도할 수 있게 되었다. 자신의 감정이나 요구를 솔직하게 표현하거나 필요한 사항을 요청할 때 상대방으로부터 공감과 위로를 얻었으며, 서로 도움을 주고 받게 되었다.

명절에 시댁에 가면 너무 힘들 것 같은데, 인사는 가야할 것 같고... 남편에게 저의 생각을 이야기해서 서로 입장을 애기하고 적당히 있다가 나오는 걸로 타협했어요. (참여자 2)

원하는 것을 요청하면 남편이 해주니까 몸과 마음이 너무 편해요. 얘기를 해주니까 서로 원하는 것을 빠르게 알고 해줄 수 있어서 좋았어요. (참여자 3)

자가격리 기간에 설이 끼어 있으니까 잠잠해지면 시간 내서 찾아 뵙겠다, 죄송하다고 이야기를 먼저 했는데, 시어머니가 나중에 시간 내서 들리면 되지, 설에 안 와도 된다고 말씀하시더라고요. 말하지 않았으면 서로 서먹하고 어려웠을 것 같아요. (참여자 5)

#### 주제3. 긍정적 삶으로의 도약

참여자들은 자신을 불행하게 만들었던 사고에서 벗어남으로써 불안정하고 부정적이었던 감정들이 해소되어가는 것을 느끼게 되었다. 일상생활에서 인지행동적 접근을 적용하게 되면서, 자신의 감정과 사고를 조절할 수 있으며 갈등 상황을 잘 풀어갈 능력이 있다는 자기 믿음이 생겨났다. 자신이 가장 소중한 존재이며 행복해야 함을 깨닫고, 보다 성공적인 삶을 꾸려갈 수 있다는 가능성을 바라보게 되었다.

##### • 감정의 치유

참여자들은 임신과 출산에 대한 책임감과 부담 때문에 부정적인 감정들로 고통스러워 했지만 프로그램에서 만난 임신 여성들로부터 공감과 유대를 형성할 수 있었다. 타인의 이야기를 들으며 같은 상황을 경험했던 자신의 경험에 비추어봤을 때 상황을 객관적으로 주시할 수 있었고, 같은 처지의 사람들로부터의 조언은 깊게 마음에 받아들여졌다. 임신의 과정 속에서 외로움과 무능함이 부정적 감정을 더욱 악화시켰지만 나만 그런 것이 아니라는 안도감에 부정적 감정이 누그러졌다.

다른 사람도 그렇다는 것을 생각하게 되고 다른 사람의 해결 능력이나 방식을 보면서 나에게 접목하면서 감정적으로 휘둘리는 게 더 적어진 것 같아요. 우울감도 줄어든 것 같아요. (참여자 1)

저도 남편을 가족의 범주에 넣고 나니 함께 할 수 있게 되었어요. 혼자 걱정하고, 외롭고, 불안한 감정이 많았는데 이제는 정도가 많이 줄어들었고, 외롭고 불안한 감정이 거의 없어졌어요. (참여자 2)

비슷한 시기 임산부의 생각을 들을 수 있고, 두려움과 불안이 나만 겪는 게 아니다고 생각하니 불안이 줄어들었어요. (참여자 5)

##### • 자신에 대한 신뢰

참여자들은 갈등 상황을 회피하고 제대로 대처하지 못했던 과거와 달리 극복해낼 수 있다는 자신감을 가지게 되었다. 더 이상 자신을 과소평가하지 않고 어떤 일이든 해낼 수 있는 능력이 있다고 다시 평가하게 되었다. 앞으로도 계속해서 긍정적인 생각을 선택하고, 과거에 집착하지 않으며, 자녀 양육을 잘 할 수 있을 것이라는 자기 믿음이 생겨났다.

아기가 커서 유치원 가면 엄마들끼리 친해야 아기들도 친하다고 하는데 나 때문에 왕따 당할까 걱정을 많이 했어요. 근데 지금은 할 수 있을 것 같아요. (참여자 2)

가보지 않은 길이 가고 싶은 거지, 굳이 시간을 돌려준다고 하면 지금 행복한데 그 때로 돌아가려고 하지는 않을 것 같아요. 현실에 잘 할 수 있다는 확신이 생겼어요. (참여자 4)

내가 생각하는 좋은 부모의 기준이 막연하구나, 최선을 다하면 어느 정도 육아를 해낼 수 있지 않나 자신감을 얻게 된 것 같아요. (참여자 5)

지금은 병원에서 조금만 안 좋다고 해도 이거는 내가 잘 해결할 수 있다, 애기는 잘 크고 있다, 내가 좌절할 필요 없다, 나는 잘 지내고 있다. (참여자 6)

##### • 행복의 기준 재정립

참여자들은 자신의 행복과 갈등의 해소를 자기 밖에서 찾았지만 진정한 행복의 기준은 자신으로부터 시작됨을 깨닫게 되었다. 갈등 상황 속에서 자신이 행복한 방향에 선택의 기준을 두고 자신에게 집중하게 되면서 비로소 마음의 평화를 얻게 되었다. 행복이 기준이 세상이나 주변 사람이라 생각했지만 이제는 자신이 기준이 되어야 다른 사람에게도 행복이 흘러갈 수 있다고 생각하게 되었다.

행복하지 않은 일에 초점을 맞추어 인생을 나락으로 떨어뜨리기보다 행복한 것에 초점을 맞춰서 업 되는 방향으로 갈 것 같아요. (참여자 1)

남편을 너무 배려만 하고 저를 안 챙겨서 그랬던 것 같아요. 나에게 수고했네. 적당히 하자라고 말하고 싶어요. (참여자 2)

행복은 절대적이 아니라 내 마음속의 상대적인 가치인 거 같아요. 내 안에 있는 최선의 행복을 느끼는 것, 내 기준에 맞춘 것이 행복이라고 생각해요. (참여자 4)

## Discussion

본 연구에서 우울한 임신 여성의 화상회의 CBT 참여 후 인지변화 경험의 의미와 구조를 탐색한 결과, 도출된 주제로는 알아차림의 연속, 회복을 위한 변화, 긍정적 삶으로의 도약으로 나타났다.

첫 번째 주제는 알아차림의 연속으로 나타났다. 참여자들은 화상회의 CBT를 통해 우울, 불안, 두려움, 열등감 등과 같은 자신의 부정적 감정을 발견하고, 부정확한 판단을 하게 만드는 사고의 문제점을 발견하게 되었다. CBT에서는 인지적 각성과 변화를 위해 특정 상황에서 일어나는 감정과 사고 및 행동의 상호작용을 다루기 때문에[23], 알아차림이 핵심적인 과정이자 중요한 요소가 된다. 자신의 감정 변화를 발견하게 되면 감정 표현과 연결된 사고의 패턴 속에서 자신이 왜곡하고 있었던 사고가 무엇인지 끌어낼 수 있게 된다[7]. 공황장애 기독교인을 대상으로 한 대면 CBT에서 참여자들은 불안, 두려움, 원망, 혼란스러움 등의 감정과 연결하여 하나님을 벌주시는 잔인한 분으로 왜곡하여 인식하는 자신을 발견하였다[15]. 강박장애 환자들은 반복적으로 떠오르는 부정적인 생각들이 자신의 비합리적 신념에서 기인했으며, 강박증이 자신의 병의 증상임을 알게 되었다[16]. 도박중독자들은 도박 충동 시 일어나는 생각을 깨달았으며, 도박이 가족에게 행복을 가져다 준다는 생각이 오류였음을 깨닫게 되었다[18]. 우울과 자살 위험이 높은 여대생들은 꼬리표 붙이기, 절대주의적 사고, 문제 해결, 당위적 사고 등 자신의 인지오류의 유형을 확인할 수 있었다[24]. CBT는 현재의 문제에 집중하여 사건, 감정, 사고, 행동을 다루기 때문에 참여자들은 현재 자신의 부정적인 감정과 증상들을 쉽게 확인할 수 있게 된다[7]. 이러한 문제지향적 접근방식은 어떤 사건과 관련하여 절망, 회피, 무기력 등과 같은 자신의 부적응적인 행동[7]에서 벗어나 새로운 관점에서 문제를 바라보는 “통찰의 경험”을 가지도록 만들 수 있다[13]. CBT에서는 내담자가 부적응적인 사고를 인식하여 이를 적응적 사고로 재구성하기 위해 치료자의 역할이 매우 중요하다[24]. 특히 부정적 패턴으로의 재발을 예방하기 위해 적극적인 자기 반성이 필요하며, 이는 치료자와의 지속적인 접근을 통해 가능하다[12]. 화상회의를 통한 CBT에서도 치료자가 실시간으로 피드백과 지원을 할 수 있기 때문에, 개별적인 사례에 대한 치료적 개입이 가능하여 내담자들이 자신의 사고에 대해 인식[7] 하도록 도움을 줄 수 있었다.

두 번째 주제는 회복을 위한 변화로 나타났다. 참여자들은 부정적인 사고의 패턴을 바꾸려고 시도했으며 대인관계의 갈등을 줄여나가기 위해 노력하였다. CBT는 개인의 인지 및 행동 패턴의 인식을 다루기 때문에 자신의 패턴을 식별할 수 있다고 생각하게 되면 변화의 동기를 가지게 된다[12]. 즉, 부정적인 사고의 수정은 행동 활성화, 즉 행동에 활력을 주고 적응적 행동을 촉진시킬 수 있는 기초가 된다[7]. 과민성 대장증후군 환자가 CBT 이후 공공화장실을 피하는 것과 같은 제한적인 행동에서 스스로를 해방시킨 것도 같은 맥락으로 이해할 수 있다[12]. Kim과 Park [24]의 연구에서도 대상자들은 자살 관련 사고를 적응적인 대안으로 변경했으며, CBT가 대안적 사고를 찾도록 사고의 유연성을 강화시키는 데 효과적이라고 하였다. 군 전역 후 학업과 진로 고민으로 우울 증상이 있는 대학생의 경우 학업과 관련된 부적응적 사고를 적응적인 사고로 변화시킬 수 있었다[25] . 도박중독자들은 도박 충동이 들 때 행동으로 옮기기 전 멈추려고 시도하게 되었고[17], 우울증 청소년에서도 CBT 원칙을 기억하고 적극적으로 일상생활에서 사용하는 것으로 나타났다[13]. Yang [15]의 연구에서는 신과 관련된 부정적 사고를 차단하기 위해 속단하기와 재앙화 사고를 통해 변화를 시도하였고, 강박장애 환자에서도 멈추기, 생각 바꾸기, 미루기 등의 다양한 대처기술을 생각하게 되었다[16]. 또한 CBT에서 대인관계의 변화는 중요한 효과 중의 하나이다[26]. Yang [15]의 연구에서도 ‘변화를 향한 도전’의 하나로 참여자들은 자신의 생각을 표현하고 도움을 요청하는 등 대인관계의 개선을 경험하였다. 강박장애 환자들은 치료 전 대인관계에 어려움이 있었으나 치료 이후 주변사람들로부터 긍정적인 표현을 듣게 되는 변화가 생겼다고 하였다[16]. 불안장애 환자에서도 타인의 부정적 평가에 대해 두려움이 있었으나 대인관계에서의 인지오류를 깨닫고 인지의 재구성 과정을 거치면서 타인에게 당당해지고 긍정적인 반응을 얻게 되었다고 하였다[27]. 참여자들은 프로그램 전반에 걸쳐 CBT 개념을 강화하고 습관을 형성하게 되었으며, 경직된 신념을 버리고 인지적 변화를 만들어낼 수 있었다[14]. 목표 설정과 문제 설정과 같은 구조화 기법과 함께 매 회기마다 일상생활에서 CBT의 개념들을 적용하는 연습을 통해 내담자는 올바른 목표를 향한 효과적인 대처 기술을 형성할 수 있게 된다[7].

세 번째 주제는 긍정적 삶으로의 도약으로 나타났다. 참여자들은 이전과 달라진 긍정적인 변화들을 경험하면서 앞으로의 삶에 대한 의지와 기대를 가지게 되었다. 화상회의 CBT 사례연구[25]에서도 대학생의 우울 증상이 감소하였고, 자신의 강점을 찾았으며 진로에 대한 구체적 계획을 세울 수 있었다고 하였다. O’Mahen 등[28]의 연구에서도 임신 여성들은 CBT가 다른 생활 방식을 채택할 수 있다는 ‘변화의 기회’라고 언급하였으며, 마음의 여유가 생기면서 삶의 질이 향상되었다고 보고하였다. Yang [15]의 연구에서는 공황장애 기독교인들은 같은 아픔을 가진 신앙 공동체를 경험하면서 연대감을 형성하고 우울이 감소하였다고 하였으며, 류마티스 관절염 환자에게 적용한 CBT 효과에서도 부정적 정서의 감소를 확인할 수 있었다[29]. 또 다른 변화로는 과거 열등감과 자기비난에서 벗어나 자신의 가치와 능력을 믿게 되었고, 자신감이 생겨났다. 이는 Hughes 등[12]의 연구에서 집단 CBT에 참여한 과민성 대장증후군 환자들이 질병에 대한 통제력이 높아지고 대처에 대한 자기효능감이 높아졌다는 결과와도 비슷하였다. 도박중독 환자들도 실수나 재발에 대한 대처능력이 향상되었으며 도박행동을 중단할 수 있다는 자신감을 가지게 되었다[17]. 또한 참여자들은 자신의 가치에 집중하며 자신을 위한 행복의 기준을 찾아가게 되었다. CBT에 참여한 강박장애 환자들도 자신의 외모에 관심이 생기고, 일상의 소소한 일에 행복을 느끼게 되었다고 하였다[16]. 마음챙김 기반 CBT에 참여했던 도박중독 환자의 경우 자신이 중요하게 생각하는 것에 우선 순위를 두고 자신의 역할과 책임에 대한 가치를 인식하게 되었다고 하였다[17]. 이와 같이 집단 화상회의 CBT 프로그램은 임신 여성의 우울 감소뿐 아니라 자기 믿음과 자신감의 향상을 통하여 더 나은 삶으로의 희망과 기쁨[11]을 가지게 하였다.

결론적으로 화상 CBT 프로그램을 통해 기존의 대면 CBT [15,16]에서와 같이 감정, 사고, 행동에서의 긍정적 변화, 왜곡된 사고 인식과 탈피, 감정과 사고의 통제, 부정적 감정의 감소, 문제 해결 능력 향상, 관계 회복, 자신감 향상 등의 변화를 확인할 수 있었다. 특히 주목할 점은 인지적 변화의 측면에서 왜곡된 사고를 효과적이며 바람직한 결과를 위한 합리적인 사고로 변화할 수 있었으며, 이러한 변화는 치료자의 개입에 의해 가능했다는 점이다. 따라서 집단 화상회의 CBT 프로그램이 COVID-19와 같은 비대면 상황에서 치료자와의 접촉을 유지하는 임신 여성의 우울관리에 새로운 중재방법임을 시사한다. 향후 임신 중 우울이 높은 여성을 선별하여 치료자에 의한 화상 CBT을 제공한다면 산후우울증을 예방하는 데 도움이 될 것이라 기대되며, 본 연구의 진술문을 수기집으로 활용하여 우울 관리를 위한 화상 CBT에 대한 이해를 높임으로써 프로그램 참여 결정을 도울 수 있을 것이다.

국내에서 화상과 같은 웹 기반 CBT 연구는 아직 초기 단계에 있으므로, 우울 관리를 위한 개입 효과를 명확히 확립하기 위하여 지속적인 연구가 필요하다. 본 연구 결과에 따라 임신 중 우울 수준이 높은 여성들을 위하여 인지 변화에 기반한 화상 통신 우울 관리 프로그램이 개발되고 적용되어야 할 것이다. 또한 본 연구는 화상 CBT의 인지적 측면에서의 긍정적 변화에 초점을 두었으므로, 치료자나 구성원과 같은 치료적 의사소통과 치료적 관계에서의 역동을 다루는 후속 연구를 제언한다.

## Figures and Tables

**Table 1.** General characteristics of participants

Characteristic	Participant No.					
	1	2	3	4	5	6
Age (year)	36	37	36	30	33	34
Job	Yes	No	Yes	Maternity leave	No	No
Number of pregnancy	2	1	2	2	1	2
Gestational age (week)	28	19	18	20	26	21
Complications of pregnancy	No	No	Yes	No	No	No
Planned pregnancy	Yes	Yes	Yes	Yes	No	Yes
Family history of depression	No	No	No	No	Yes	No
EPDS score						
Before CBT	20	12	14	11	12	17
After CBT	5	5	3	7	7	6

CBT: cognitive behavioral therapy; EPDS: Edinburgh Postnatal Depression Scale.

**Table 2.** Experience of cognitive changes in cognitive behavioral therapy using video communication for depressed pregnant women

Theme	Subtheme	Code
Continuing realizations	A negative and instable self	Having emotions such as depression, lethargy, or anxiety
		Having severe mood swings and becoming carried away by emotion
		Avoiding conflict situations
		Blaming oneself in negative situations
	A selfish judgment that excludes others	I decide everything on my own so as not to harm others.
		I was sacrificed by marruage and childbirth.
		Those who disagree with me do not understand me.
		A boss who gives pregnant women complicated tasks is a disrespectful person.
	A strong belief in self-control	I must not harm others.
		Having to do my job perfectly by myself
		Having to be successful in order to be recognized by others
		Being nice to everyone
Attempt to change for restoration	Shift to rational thinking	Finding valid reasons in emotional situations
		Taking time to contemplate without expressing one’s feelings immediately
		Finding alternatives without focusing on emotions
	Freedom from suppressed beliefs	Not having to sacrifice oneself for others
		Anyone can make a mistake
		Recognition of others is not necessarily important
		Not having to bear everything oneself
	Tolerance of other people	Understanding the weight of the responsibilities and the difficulties at work that my husband experiences
		Like me, my husband is experiencing pregnancy for the first time.
		Judging my husband’s passive personality badly by my standards
		Understanding the position of the other person, who is my boss
	Courage for self- expression	Expressing my feelings and asking others to talk first
		Listening to others and balancing each other’s needs
		Asking others for help
		Explaining to others the reason for my behavior and asking for forgiveness
Departure for a positive life	Emotional healing	Reduction of depression
		Reduction of anxiety
		Reduction of anger
		Reduction of psychological burden
	Faith in oneself	Controlling my emotions and thoughts
		Resolving interpersonal relationships
		Focusing on reality rather than clinging to the past
		Parenting confidence
	Reestablishing the criteria for happiness	Focusing on my own happiness and rewards and encourage myself
		The standard of value in on the inside, not the outside.
		The standard of happiness is me, and I will make it.
